# Using a Motion Sensor to Categorize Nonspecific Low Back Pain Patients: A Machine Learning Approach

**DOI:** 10.3390/s20123600

**Published:** 2020-06-26

**Authors:** Masoud Abdollahi, Sajad Ashouri, Mohsen Abedi, Nasibeh Azadeh-Fard, Mohamad Parnianpour, Kinda Khalaf, Ehsan Rashedi

**Affiliations:** 1 Department of Industrial and Systems Engineering, Rochester Institute of Technology, Rochester, NY 14623, USA; ma8489@rit.edu (M.A.); nafeie@rit.edu (N.A.-F.); 2 College of Business and Law, RMIT University, Melbourne VIC 3000, Australia; ashouri.sajad@gmail.com; 3 Department of Physiotherapy, Physiotherapy Research Center, School of Rehabilitation, Shahid Beheshti University of Medical Sciences, Tehran 1616913111, Iran; mohsenabedi110@sbmu.ac.ir; 4 Mechanical Engineering Department, Sharif University of Technology, Tehran 1136511155, Iran; parnianpour@sharif.edu; 5 Department of Biomedical Engineering and Health Engineering Innovation Center, Khalifa University of Science and Technology, P.O. Box 127788 Abu Dhabi, UAE; kinda.khalaf@ku.ac.ae

**Keywords:** objective clinical decision-making, wearable systems, trunk kinematics, pattern recognition, classification, STarT back screening tool

## Abstract

Nonspecific low back pain (NSLBP) constitutes a critical health challenge that impacts millions of people worldwide with devastating health and socioeconomic consequences. In today’s clinical settings, practitioners continue to follow conventional guidelines to categorize NSLBP patients based on subjective approaches, such as the STarT Back Screening Tool (SBST). This study aimed to develop a sensor-based machine learning model to classify NSLBP patients into different subgroups according to quantitative kinematic data, i.e., trunk motion and balance-related measures, in conjunction with STarT output. Specifically, inertial measurement units (IMU) were attached to the trunks of ninety-four patients while they performed repetitive trunk flexion/extension movements on a balance board at self-selected pace. Machine learning algorithms (support vector machine (SVM) and multi-layer perceptron (MLP)) were implemented for model development, and SBST results were used as ground truth. The results demonstrated that kinematic data could successfully be used to categorize patients into two main groups: high vs. low-medium risk. Accuracy levels of ~75% and 60% were achieved for SVM and MLP, respectively. Additionally, among a range of variables detailed herein, time-scaled IMU signals yielded the highest accuracy levels (i.e., ~75%). Our findings support the improvement and use of wearable systems in developing diagnostic and prognostic tools for various healthcare applications. This can facilitate development of an improved, cost-effective quantitative NSLBP assessment tool in clinical and home settings towards effective personalized rehabilitation.

## 1. Introduction

Considered as the most common cause of disability, as well as the most widespread musculoskeletal disorder worldwide, low back pain (LBP) remains a tremendous health and socioeconomic challenge of pandemic proportions [1,2]. It is estimated that around 84% of the global adult population will experience LBP at least once during their lifetimes, where every second, up to 33% adults, or one in every three people, suffer an LBP episode [3,4,5]. In both developed and developing countries, LBP has been identified as the fourth-most frequent condition warranting a visit to a physician, the fifth-most common reason for hospitalization, and the third-most frequent cause for surgery [4,6]. Meanwhile, about 90% of LBP patients are identified as nonspecific low back pain (NSLBP) patients, or LBP patients with no recognizable specific pathology [7]. There is, therefore, a compelling need for investigating this highly prevalent condition and leveraging readily. 

Meanwhile, researchers confirm that there is no “one-size-fits-all” management approach for NSLBP patients, given the elusive etiology, complexity, and trigger heterogeneity among sufferers [8,9]. Evidence-based guidelines and outcomes, on the other hand, emphasize the necessity of stratifying NSLBP patients in primary care according to (a) risk for subsequent disability, and (b) specific treatment approaches [10,11]. While this may seem straightforward, it is quite challenging to implement. Indeed, several methods have been proposed towards improved NSLBP patient classification [12,13,14,15]. One of the most commonly accepted and reliable available instruments for stratifying treatment based on prognosis risk is the STarT Back Screening Tool (SBST) [12,16,17,18,19]. This approach requires NSLBP patients to complete a screening questionnaire, the results of which are used to assign the respondent into one of three groups: low, medium, and high risk [20], with specific guidelines prescribed for each of the three groups. Notably, high-risk ranking requires the patient to undergo a series of six individual physiotherapy sessions over three months, which significantly differs from the proposed treatment for the other risk groups, both in terms of dose and type of therapy. The SBST approach focuses on reducing pain and disability, along with improving psychological functional state of mind, by means of combined physical and cognitive-behavioral therapies. In contrast, low- and medium-risk groups are typically treated with less demanding strategies, including providing therapeutic information to low-risk patients, and suggesting a brief course of physiotherapy for medium risk patients. It is, therefore, critical to distinguish the high-risk individuals among NSLBP patients, in order to allow for timely much-needed targeted intervention.

Earlier studies have confirmed that stratifying patients into different groups and prescribing appropriate treatment leads to improved therapeutic outcomes (e.g., decreasing NSLBP disability and reducing time off work) [21]. Clinical decision-making in primary care is currently mostly based on qualitative questionnaires. On the other hand, recent advances in wearable sensor technology provide new opportunities to develop quantitative assessment methodologies, such as capturing and analyzing patients’ movements during walking or other daily life activities [22,23,24,25,26]. The underlying principle behind these studies centers around utilizing variability in trunk motion (i.e., trunk kinematics) to assess and discriminate people suffering from NSLBP.

A great deal of studies have investigated kinematic-based approaches for diagnostic and clinical decision making in primary care [22,27,28,29,30,31,32]. Earlier studies involving LBP sufferers have confirmed a correlation between the quality of motion in people and their health status [33,34,35,36]. The main hypothesis in these studies assumes that LBP instigates changes in trunk kinematics. Marras et al. implemented several models to classify healthy versus NSLBP subjects, based on trunk angular motion features during trunk flexion/extension and bending, in various symmetric and asymmetric planes of motion [34,37]. Marras et al. implemented the same approach to classify the LBP patients according to the anatomic aspects and pain locations [34]. In our earlier study, we were also able to distinguish healthy individuals from LBP patients based on measurements obtained from an inertial measurement unit (IMU) sensor placed on the trunk [22]. In addition to LBP, similar studies have focused on the neck region or cervical spine. For example, Bahat et al. revealed that neck pain could lead to lower peak and mean velocity of the neck during flexion/extension movements [38]. This suggests that pain in any particular musculoskeletal region could alter motion.

All of the above-mentioned studies, and many more, have sought to explore models that could distinguish NSLBP patients from their healthy counterparts. By contrast, far fewer scholarly works aimed to stratify NSLBP patients using kinematic data [34], particularly in alignment with clinical guidelines and treatment options [39]. The purpose of this study was, therefore, to use AI techniques to investigate the categorization of NSLBP patients based on kinematic data obtained from wearable sensors, balance-related measures, and psychosocial parameters, in conjunction with STarT output. We postulate that various aspects of the STarT questionnaire, including psychological features, are reflected in altered human kinematics/motion, and hence recording that motion inherently captures these features. This study is expected to facilitate the objective assessment and categorization of NSLBP, which may help clinicians and healthcare providers make better informed decisions regarding patient risk levels, towards effective targeted diagnosis and treatment.

## 2. Materials and Methods

**Figure 1:**
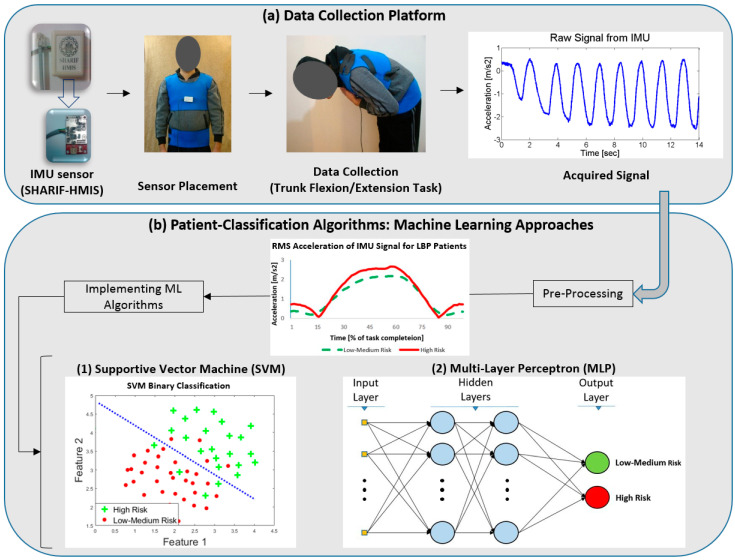
Schematic system diagram of the platform for the classification of NSLBP (nonspecific low back pain) patients. (**a**) Sensor and data collection procedure. (**b**) Data analysis procedure.

The system diagram provided here depicts the overall protocols utilized for data collection and analyses in this study (Figure 1). Details about each aspect are described in the following sections.

### 2.1. Participants

Ninety-four male volunteer NSLBP patients were recruited for this study. The average (standard deviation) age, height, and weight of the participants were 43.6 (6.9) years, 172.6 (7.3) cm, and 79.5 (12.5) kg, respectively. There were no significant differences among the groups in terms of age, height, weight, and body mass index (BMI) (Table 1). According to the results of the STarT questionnaire, the number of high, medium, and low risk patients were 28, 37, and 29, respectively. Three inclusion criteria were implemented: (1) Participants, between the age of 20–50, had to be identified with nonspecific low back pain with or without radiating leg pain [40]; (2) During the assay, pain intensity had to remain less than five on the Visual Analog Scale (VAS) [41,42]; and (3) All included participants had to be free of any form of spinal surgery. The NSLBP patients were examined by an orthopedic surgeon to meet the inclusion criteria, and participation was voluntary. Each individual was also required to sign an informed consent form, approved by the Shahid Beheshti University of Medical Sciences Ethics Committee, prior to taking part in this study.

### 2.2. Experimental Design

Each subject was asked to perform as many trunk flexion/extension movements as he comfortably could in the sagittal plane, within a 14-s time period [22]. They were instructed to start the movement from standing in an upright relaxed position. An IMU sensor (9DOF Razor IMU, Sparkfun^®^, Niwot, CO, USA), composed of a 3-axis accelerometer and a 3-axis gyroscope with sampling frequency and full-scale range of 20 Hz and ±2000 °/s was utilized to acquire trunk kinematic data. The sensor was placed on the sternum of the patients during movement, based on literature [22,34,37,43] (Figure 1), to track the linear acceleration and angular velocity. The sensor collected the data in three directions of X, Y, and Z, which were defined as Vertical (proximal-distal), mediolateral, and anteroposterior directions, respectively.

In addition to the kinematic data, a Wii Balance Board (Nintendo^®^, Kyoto, Japan), with the same function of a typical force plate was used to record center of pressure (COP) data. This signal was employed to calculate balance-related measures while the participants performing trunk flexion/extension. The sampling frequency and full-scale of the board were 40 Hz and 150 kg respectively. Moreover, each participant was required to complete the Hospital Anxiety Depression Scale (HADS) [44], as well as, the Tampa Scale of Kinesiophobia (TSK) [45] questionnaires. These quantitative measures were implemented to analyze if the kinematic data, in combination with other types of information (balance board data and psychosocial factors), can yield better results. Finally, the subjects were asked to complete the translated version of the STarT questionnaire [46], which divided (labeled) the NSLBP patients into three risk groups (low, medium, and high). The output of STarT was implemented as ground truth input in our machine learning algorithms.

### 2.3. Data Processing

#### 2.3.1. Data Preparation

For each subject, the collected data were processed using in house custom codes written in MATLAB (Mathworks, Inc., Natick, MA, USA) to provide feature vectors for classification. Since the participants were instructed to perform flexion/extension as fast as they comfortably could within a specific time period (i.e., 14 s), the number of cycles varied from one person to another. Therefore, for each participant, each sensor signals were aggregated into one cycle, in order to produce a single motion profile representing the kinematic features of the reported signal. This step led to determining the angular/linear acceleration and angular velocity profiles for each subject in only one resultant cycle. We segmented the flexion/extension cycles using the X-axis gyroscope data. Filtration of the X-axis gyroscope (which is in line with flexion/extension axis) data with a very low frequency of 1 Hz resulted in obtaining the kinematic signal for the main flexion/extension movement. Given the points that angular velocity in X-axis alter from negative value to positive value, starting points of each cycle were identified. As all of the sensor data are harmonized in terms of the time of measurement, we selected the identified points in the signals of the remaining gyroscopes and accelerometers. We, therefore, segmented the identified cycles accordingly. Since these cycles could last for different amounts of time, they were time-scaled to obtain 101 data points from each cycle. The resultant signal was then calculated by averaging (using the root mean square) all of the associated points in these cycles, given Equation (1):

(1) $$C_{i}=\sqrt{\frac{C_{i1}{}^{2}+C_{i2}{}^{2}+C_{i3}{}^{2}+\ldots +C_{in}{}^{2}}{n}}$$

where, $C_{i}$ is the *i*th point in the aggregated cycle, $C_{ij}$ is the *i*th data point of the *j*th cycle, and n is the number of cycles in the signal. For example, to calculate data point number 50 in the resultant signal, the root mean square of the data point 50 in all of the cycles was calculated. Since the initial point for each segment was the same, it was excluded from feature vectors, and consequently each signal included only 100 data points. Therefore, the time variable was eliminated from the data, and the resulting data represented the kinematics profiles in each percentile of task completion. The segmentation, time-scaling, and aggregating processes was performed on all the components (x, y, and z) of the linear/angular acceleration and angular velocity signals. Finally, the signals were concatenated to have a single dimension signal, which included all signals together.

#### 2.3.2. Feature Selection

This study included four performance-oriented types of features: (1) linear acceleration, (2) angular velocity, (3) angular acceleration, and (4) force platform data. The angular acceleration was derived from angular velocity before segmentation and time-scaling. It should be noted that by removing the time variable from the signals, through time-scaling and averaging the cycles, the effect of the different number of cycles for each trial could be eliminated. Hence, the main reason for adding angular acceleration was reflection of important changes in the angular velocity due to the difference in the number of cycle among the participants. To shed light on this point, if we assume two different signals for angular velocity—a single cycle and four cycles, the resultant signals would be the same as long as they have the same range and trend of angular velocity. However, the range of angular acceleration corresponds to the number of cycles for each subject, such that more flexion/extension cycles during a fixed period of time (i.e., 14 s) result in a wider range of angular acceleration.

As to Table 2, we employed four feature sets in this study:

- Full signal features including angular velocity, linear acceleration and angular acceleration in three directions of X, Y and Z. This feature set resulted in 900 features.
- Sixteen significant statistical features from the variables in the full signal, as they are described in Table 2. For example, frequency at maximum power indicates the frequency associated to the maximum power in spectral analysis of the signal and median frequency estimates the median normalized frequency of the power spectrum of the signal. It is noteworthy that these 16 scales were calculated for each of the variable (angular velocity, linear acceleration and angular acceleration) in each direction (X, Y and Z). Hence, each feature vector has the length of 144 (16 × 3 × 3).
- Balance analysis features that were extracted from the COP data for the whole duration of the experiment. These four measures obtained from the force platform placed underneath the participants’ feet, namely, the COP range of displacement in both x and y directions, the COP path length during the motion, and the ellipse area of the COP’s amplitude which was area of the motion ellipse, which captured 95% of the COP data [47]. These features enabled the algorithm to consider balance factors as a part of the discriminating protocol.
- Subjective features included HADS and TSK questionnaire.

Finally, to avoid the bias of the difference of feature types, all of the features were scaled to have an absolute value of ≤1 scale variance prior to applying any machine learning algorithm. To this end, the following equation was implemented:

(2) $$X_{ij\;new}=\frac{X_{ij}-\bar{X_{j}}}{\sigma_{j}}$$

where, $X_{ij\;new}$ is the value of the normalized feature, $X_{ij}$ is the measured value for the feature, $\bar{X_{j}}$ is the average value of that feature for all the subjects, and $\sigma_{j}$ is the variance of each observation.

### 2.4. Classification Approaches

After data processing, a total of three AI algorithms were implemented in MATLAB to classify the patients into distinct risk groups. First, the K-means algorithm was applied to cluster the participants based on their kinematic data without the use of any labels (unsupervised learning). According to a recently published review paper, the two common supervised machine learning approach in studies related human movement are support vector machine (SVM) and Neural Network (NN) [48]. Hence, the data was evaluated using a SVM and NN algorithm. To determine the best discriminating approach, the feature set of FS (i.e., full signal, see Table 2) was implemented in all the algorithms.

#### 2.4.1. K-Means

K-means is an unsupervised learning method used to cluster data without labeling. In this study, we used K-means to (1) check how many subgroups could appropriately represent all NSLBP patients based on trunk kinematics, and (2) assess if the kinematic data was properly classified. The latter objective intended to obtain general observations about the kinematic behavior of the patients. This algorithm generally starts at initial/center points (equal to the number of clusters), and then systematically adds each point to one of the clusters, based on its distance from center points (calculated by the squared Euclidean distance metric). After each iteration of assigning the data points to their relevant clusters, the center points of the clusters update according to the latest results. This process continues until the center points converge to their final values. Finally, all of the sample data is assigned to one of the clusters. Since the K-means algorithm uses the optimal number of clusters as input, it is critical to find this number a priori.

Here, the proper clustering number was determined based on the Calinski–Harabasz index. It was calculated for different kinematic features, including the angular velocity, linear acceleration, and combination. The formulation of the index was as follows [49]:

(3) $$\mathrm{Calinski}-\mathrm{Harabasz}\;\mathrm{index}=\frac{SS_{B}}{SS_{W}}\times \frac{N-k}{k-1}$$

where $SS_{B}$ is the overall between-cluster variance, $SS_{W}$ is the overall within-cluster variance, *N* is the total number of observations, and *k* is the number of clusters. The number of clusters was determined such that this index could attain the maximum possible value. The first ratio illustrates the case of having more variance between clusters yet less variance in each cluster, thus causing the index to increase. (i.e., the index increases if the clusters are far away from each other).

#### 2.4.2. Support Vector Machine (SVM)

The SVM method was utilized to see how the labeled data could be discriminated using a supervised learning algorithm. Specifically, a leave-one-participant-out cross-validation was employed, which is typically useful when the sample size is not large. Specifically, in this approach, during each iteration, the method excludes the data from one participant and is trained based on the rest of dataset. Then, the trained model predicts the label of the excluded participant according to his/her input. Comparison of the predicted and true labels determine the accuracy of the model. The kernel function was selected based on the best outcome among linear, polynomial, and Gaussian radial basis kernel functions. According to [22], we tried three regularization parameters (C), i.e., (0.1, 1, 10) along with second-degree for polynomial function and also three RBF kernel scales, i.e., (0.1, 0.5, 1) for the RBF function. The SVM algorithm was implemented for each of the subjects, such that all other subjects were considered for training data in that particular run. The algorithm then predicted the label of the patient using the relevant trained network.

Initially, multi-class SVM was used to segregate patients into the three risk groups: low, medium, and high. Since the accuracy of the three-class SVM approach for all feature combinations was less than 40%, we combined two groups and compared them to the third one. To this end, the SVM algorithm was run to calculate the accuracy, sensitivity, and specificity of the method based on comparing the predicted label with the ground truth.

Considering that the SVM method is a distance-based approach, the balance of a particular dataset could make a significant difference in the accuracy of discrimination. Since the 3 group patients were stratified into two subgroups, the sample size for the first group (Low–Medium risk) was almost twice the size of the second (High risk). To address this, sample data was randomly selected from the larger group. For example, in addition to the 28 high-risk patients, another 28 patients were randomly selected from the pool of 56 low-medium risk patients. This procedure was repeated ten times, and the model was implemented to avoid the effects of random biased sampling. We also explored other machine learning tools using the same dataset to compare the data. The results of these analyses are reported as mean and standard deviation of the ten runs.

#### 2.4.3. Neural Network

Multi-layer perceptron (MLP), a class of feedforward artificial neural network (ANN) algorithm, was also implemented in this work. For each subject, 900 features (FS feature set) were considered as the input layer neuron. The architecture of the network was designed with eight hidden layers of 700, 500, 300, 100, 50, 15, 10, and 5 neurons, respectively. These values were determined based on a manual trial-and-error process to increase the accuracy of the model. The system included a 10-layer feedforward network, where a sigmoid function was used for the hidden layers, while a softmax transfer function was applied for the output layer. Similar to the SVM model, we have implemented the leave-one-participant-out cross validation to evaluate the accuracy of the MLP model. The algorithm was run for a three-class classification. Similar to the SVM approach, the accuracy for all feature combinations did not exceed 40%; we opted for 2-class classification.

### 2.5. Performance Evaluation

The performance of the machine learning models were calculated by comparing the predicted label of the patients with ground truth data. The performance measures in binary classification of high (H) vs. low-medium (LM) risk are defined according to [50,51] as follows:

(4) $$Accuracy=\left(\frac{TP+TN}{TP+FP+TN+FN}\right)\times 100\%$$

(5) $$Sensitivity\;or\;Recall=\left(\frac{TP}{TP+FN}\right)\times 100\%$$

(6) $$Specificity=\left(\frac{TN}{TN+FP}\right)\times 100\%$$

(7) $$Precision=\left(\frac{TP}{TP+FP}\right)\times 100\%$$

(8) $$F1-score=2\times \left(\frac{Sensitivity\times Precision}{Sensitivity+Specificity}\right)$$

(9) $$G-index=\sqrt{{\left(1-Sensitivity\right)}^{2}+{\left(1-Precision\right)}^{2}}$$

where true positive (TP) is the number of patients labelled as high risk correctly, true negative (TN) is the number of patients labelled as low-medium correctly, false positive (FP) is the number of patients labelled incorrectly as high risk, and false negative (FN) is the number of patients labelled incorrectly as low-medium risk. Accuracy indicated the ability of the model to recognize the label of the patients correctly. Sensitivity and specificity are the ability of the model to recognize high and low-medium risk, respectively. F1-score is the harmonic mean of the sensitivity and precision. G-index represents the Euclidean distance between the [01] point on the receiver operating characteristic (ROC) curve, which associates with the best classifier, with the point associated with our model. In order to evaluated the model according to the G-index, the following categorization is implemented [51]: (i) optimum if G ≤ 0.25; (ii) good if 0.25 < G < 0.70; (iii) random if G = 0.70; and (iv) bad if G > 0.70.

As mentioned earlier, all three machine learning algorithms, i.e., SVM, MLP, and K-means, were run for the FS feature set. However, the approach yielding the highest accuracy was utilized for all feature combinations. Accordingly, a full factorial combination of the four feature sets ($2^{4}-1=15\;\mathrm{cases}$) was analyzed. Comparison of accuracy, sensitivity, and specificity for all cases produced the feature set(s) with the highest accuracy.

## 3. Results

**Figure 2:**
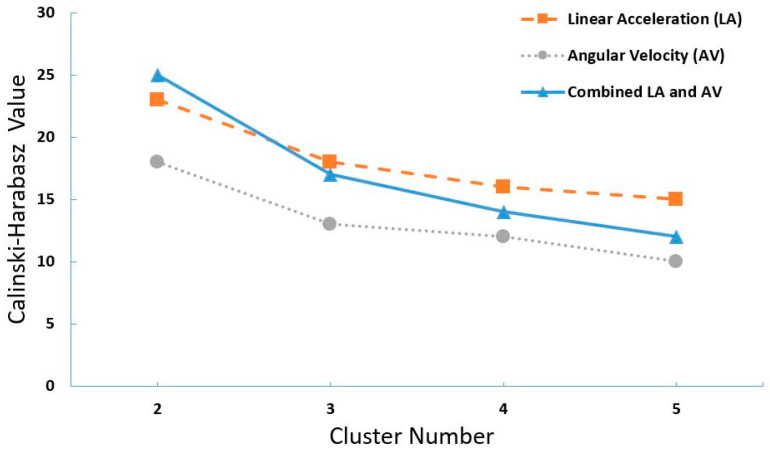
Calinski–Harabasz index for different features including linear acceleration, angular velocity, and the combination of them, for different number of clusters.

Analyzing the Calinski–Harabasz index for different features (i.e., angular velocity and linear acceleration) revealed that the maximum value for all the plots (representing different cluster numbers) occurred for the 2-cluster formulation (Figure 2). In other words, by clustering the data into two groups, we achieved the most significant variance between the clusters, as well as the lowest variance within each cluster. To evaluate the efficiency of the k-means algorithm, each cluster was labeled based on the largest sample population. For example, assuming 40 samples in the first cluster, where 34 samples had an ‘A’ label, the entire cluster would be labeled as the ‘A’ group. Accordingly, accuracy, sensitivity, and specificity for the K-means algorithm were calculated as 57%, 43%, and 63%, respectively.

After implementing the SVM and MLP algorithms for 2-class data classification accuracy, sensitivity, specificity, F1-score, and G-index of the various approaches were calculated for all possible 2-class categories (see Table 3). Out of the different combinations for the optimal regularization parameter and RBF kernel scale, the pair of (1, 10) yielded the highest accuracy in SVM models. As noted earlier, the sample size for each of the two selected classes was made equal, which required a random selection of samples from the larger group (among the two classes). This process was repeated ten times to avoid biased selection. Hence, the numbers reported here are values of the means for the ten runs and their associated standard deviation values (Table 3).

Since using the SVM approach for discriminating high vs., low-medium risk NSLBP groups yielded the highest performance, the same algorithm was run for the full factorial combination of the feature sets to evaluate the effects of different types of features on the accuracy of the model (Table 4).

## 4. Discussion

This study aimed to design and develop a machine learning-based model with the capability of classifying NSLBP patients into different risk groups identified by STarT questionnaire outcome data based on the combination of quantitative trunk kinematic data obtained from an IMU sensor and other feature sets such as FT16, Wii, and ADT. We hypothesized that the outcomes of the STarT questionnaire, largely considered as the clinical golden standard tool for LBP assessment in clinical settings, are partially reflected by the variability in trunk kinematics during flexion/extension tasks, and hence can be used to categorize patients a priori.

Initially, an unsupervised algorithm was implemented in order to evaluate the separability of the data. After determining the optimal number of groups (i.e., two classes), two different supervised machine-learning algorithms (SVM and MLP) were utilized. When we compared the outcomes of these two methods, it was clear that the SVM model delivered the highest accuracy for classifying NSLBP patients, with a maximum accuracy and lowest G-index level of 75.4% and 0.35, respectively (Table 3). Finally, using the SVM approach, other feature sets, such as FT16, Wii, and ADT (see Table 2), were added to the feature vector to investigate if the accuracy of the model could be improved by adding more information to the input data set. Our accuracy-related findings for the full factorial combination of the feature sets confirmed that the IMU full signal (FS feature set) remained the best feature for identifying NSLBP patients (Table 4).

As previously noted, we had originally planned to include the three typically identified NSLBP risk categories of low, medium, and high risk. However, based on the unsupervised machine learning approach employed here, the optimal number of groups was identified as two and not three. This was supported by the supervised machine learning results, which yielded moderate accuracy for three-class classification. Thus, based on our earlier discussion regarding the clinical value of discriminating the high-risk NSLBP patients, we opted for a 2-class classification. This decision was further bolstered by the lack of significant differences in treatment between the low and medium risk groups, as identified by our clinical STarT questionnaire results [20]. Interestingly, outcomes from the SVM model also demonstrated the substantially better discriminative capability of the motion-sensor signal in distinguishing patients with high risk vs. low-medium risk (Table 3). Further work is warranted to identify relevant delineations within the low-medium risk groups and to devise more sensitive tools and technologies to capture them.

In order to avoid the confounding effects of demographic data variability, statistical analysis, i.e., *t*-test, was performed on the data of all combinations of 2-class classification (Table 5). The results indicated no significant differences in age, height, weight, or BMI among the various groups in 2-class classification.

It is noteworthy to mention that a recent study was successful at discriminating between healthy subjects and lower back pain patients based on trunk kinematics [22]. However, the differences in trunk kinematics between the patient group and the healthy cohort were more significant, in comparison to those within the NSLBP group. In general, classifying subjects into healthy and NSLBP groups could be performed more accurately, as compared to subgrouping the NSLBP patients into different risk levels. It should also be kept in mind that there always remains a gray zone within the reference method, the STarT questionnaire in this study, in terms of appropriate group assignment for a particular patient. For example, according to the STarT questionnaire, if a patient’s score is less than 4, s/he would be assigned to the low-risk group. The challenge, however, of the extent to which can the questionnaire successfully assign patients “on the cusp” to the proper group, remains elusive. The uncertainty in labeling “cusp patients” to one group or another could also introduce inaccuracies in the results obtained from motion-based classification. Consequently, less accuracy was in fact anticipated in stratifying the NSLBP patients, as compared to discriminating between healthy and NSLBP groups.

Our results demonstrated that the accuracy of the K-means clustering was not sufficiently high, most likely reflecting the lack of clear data boundary between the high and low-medium risk groups. This analysis highlights the need to implement supervised algorithms to develop models with higher accuracy. Since we found no other existing studies which classified NSLBP patients, we used machine-learning-based models developed for other forms of clinical decision making for comparison [52,53,54]. The accuracy for the best machine learning model (i.e., the SVM algorithms) obtained in this study was in the range of clinical decision-making models, based on IMU signals or other body-worn sensors, as described in literature (<80%) [52,53,54,55]. However, G-index of 0.35 demonstrates that the model is not yet optimal (optimal model: G-index < 0.25) and could be improved in future. As compared to the SVM model, the MLP approach implemented here yielded lower accuracy (~60%), which may be due to the fairly small sample size in this study (94 for the 3-class and 56 for the 2-class classifications). Other analogous studies have indeed included a greater number of observations for each class [56,57], particularly when implementing a neural network approach [58].

The accuracy of the SVM model was investigated in connection with different feature sets. We confirmed that the COP data for the Wii feature was inadequate for discriminating between high vs. low-medium risk NSLBP patients (accuracy of ~40%). However, the ADT feature set (which included psychological information, such as anxiety and depression) achieved better results. The FT16 feature set, however, which included 16 statistical measures of the IMU signal, led to better accuracy (~67%), and specificity (~76%), as compared to the ADT. On the other hand, both demonstrated roughly the same capability in identifying high-risk subjects (sensitivity of ~56% and ~58% for ADT and FT16, respectively). Overall, the kinematic feature set of the FS was by far better than the others individually, or in combination with the FS itself (see Table 4). Combining the FS and ADT feature sets, however, did not increase the accuracy of our model. This possibly suggests that the kinematic data had already been incorporated through the psychosocial factors included in the ADT feature set, as postulated earlier. Hence, despite STarT questionnaire outcome which does not clarify the difference among different risk group in terms of physical performance and mental aspects, our results unveiled that out of all combinations of the trunk kinematic, balance measures, and psychosocial parameters, trunk kinematic was the factor that could categorize the patients in different risk groups. While balance and other measures can be important, our study has shown trunk kinematic had the largest influence on the patients’ risk labels. This knowledge could facilitate development of more targeted and effective intervention for the patients.

The inclusion criteria used in this study regarding the pain level during the experiment is noteworthy. In consultation with clinicians, we used a pain level of less than 5, based on the VAS score during trunk flexion/extension. This criterion was selected to ensure the safety of patients and prevent aggravation of their conditions. However, it may have led to the elimination of many patients who belonged to the high-risk group, hence, limiting this group to include many patients in the previously discussed grey zone (between the high and medium risk groups). This could adversely affect the results of this study and decrease the efficiency of the proposed model. Future work can benefit from adding other pain scales and neuroimaging modalities to provide additional clarity in this regard.

**Figure 3:**
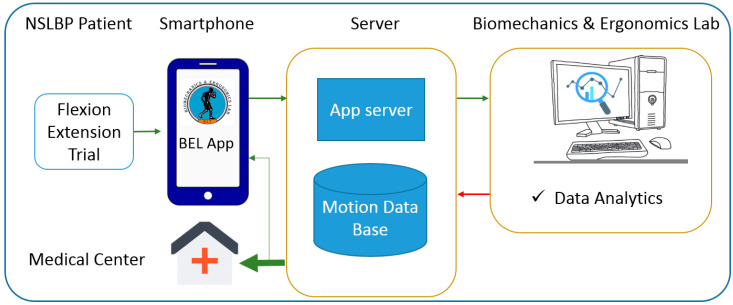
Schematic illustration of the data path in real-time NSLBP classification.

In current clinical settings, the risk status of NSLBP patients is assessed largely based on the subjective STarT questionnaire, where a qualified healthcare provider must analyze the results in order to prescribe appropriate treatment/rehabilitation protocols. The approach presented here only utilized the data from one motion sensor during trunk flexion/extension in the sagittal plane. The setup was intentionally designed as such, in order to render data collection as simple and quick as possible with some training, without the help of an expert. Such methodology can hence be implemented on an application and installed on today’s widely used smartphones, which are already ubiquitous and expected to further grow in sophistication [59,60]. As a future direction the present study, this will facilitate the data collection from a large pool of NSLBP patients remotely which in turn may increase the accuracy of the model to be implemented in clinical setting. Furthermore, it provides the patients with a real-time analysis platform during which the kinematic data will transmit to the server instantly. Having access to this data, we would be able to implement the trained model and send the results, patient’s label, to the smartphone along with the medical centers to keep the clinic updated about the patient’s status (Figure 3). However, several steps need to be taken to have such a practical tool. For example, the effects of the orientation and specification of the smartphone on the results must be investigated.

It is important to acknowledge the challenges/limitations encountered here, and suggest possible mitigation. Due to hospital policy, all the participants in this study were males, hereby eliminating gender-balanced results and conclusions. Furthermore, the hyper-parameters in MLP approach were tuned through a manual trial-and-error process. However, formal hyper-parameter tuning is recommended for further studies. Another major limitation pertains to the sample size, which based on the results may have affected the machine learning analyses. Accordingly, a follow-up investigation should recruit a larger sample size, to enhance the generalizability of the findings and the accuracy of machine learning algorithms. Investigating patient clustering during treatment also represents another interesting opportunity for future research. Specifically, investigating the impact of various treatment protocols on patient kinematics and/or self-reported clinical questionnaire data is warranted in future work.

## 5. Conclusions

This study explored the capability of using a simple cost-effective motion-capture sensor, in conjunction with STarT—the current gold standard clinical assessment NSLBP questionnaire—as a potential quantitative decision-making tool in clinical settings and telemedicine/rehabilitation applications. Our results demonstrated that machine learning methods, especially SVM, can distinguish high vs. low-medium risk NSLBP patients with an accuracy of >75%. This study also revealed that the STarT questionnaire could implicitly be captured partially by monitoring trunk motion. The findings from this investigation could facilitate the development of objective NSLBP assessment tools, with potential diagnostic and prognostic value, by leveraging modern hardware and software technologies. Closing the loop by developing an application for today’s widely available smartphones and sharing quantitative patient data with clinicians can open the door to a wide spectrum of healthcare applications with the long-awaited benefits of personalized precision medicine.

## Figures and Tables

**Table 1:** Statistical analysis on demographic data from three risk groups. The numbers in parenthesis indicated the standard deviation.

	Low-Risk	Medium-Risk	High-Risk	*p*-Value (ANOVA)
**Age (year)**	43.8 (8.2)	43.4 (5.6)	43.6 (7.3)	0.98
**Height (cm)**	172.4 (8.3)	173.7 (6.7)	171.5 (6.9)	0.47
**Weight (kg)**	80.3 (12.5)	80.5 (12)	77.5 (13.3)	0.6
**BMI (kg/m^2^)**	26.9 (3)	26.7 (3.8)	26.2 (3.4)	0.74

**Table 2:** The description of various features and their sources.

Feature	Source	Description
**Full Signal (FS)**	IMU Sensor	Linear/angular acceleration and angular velocity in X, Y, and Z directions (all time-scaled to have 100 data points)
**Sixteen Significant Features from variables in FS (FT16)**	IMU Sensor	Max, min, range, mean, quartiles, interquartile range (IQR), IQR divided by median, standard deviation, kurtosis, skewness, entropy, power, frequency at maximum power, and median frequency of the signal
**Balance Analysis (Wii)**	Balance Board	From COP data; x and y range (balance board’s axes), path length, and area of the ellipse which could capture 95% percent of the data
**Subjective Features (ADT)**	Questionnaires	Participants filled out HADS and TSK questionnaires

**Table 3:** The accuracy, sensitivity, specificity, F1-score, and G-index of the different combinations of the classes for SVM (support vector machine) and MLP (multi-layer perceptron) algorithms.

		Low vs. Medium-High	Medium vs. Low-High	High vs Low-Medium
SVM	Accuracy	46.3 (6.3)	45.5 (6.8)	75.4 (4.2)
	Sensitivity	45.0 (10)	59.7 (7.5)	72.5 (3.8)
	Specificity	47.6 (8.6)	31.4 (7.2)	78.2 (5.3)
	F1-score	45.6 (12.1)	52.3 (10.4)	74.6 (14.4)
	G-index	0.76 (0.19)	0.8 (0.15)	0.35 (0.09)
MLP	Accuracy	51.2 (4.8)	45.4 (6.3)	61.8 (5.7)
	Sensitivity	42.8 (8.7)	44.8 (8.9)	66.2 (7.1)
	Specificity	59.6 (7.2)	46.1 (7.6)	57.5 (7.3)
	F1-score	46.7 (12.7)	45.1 (11.6)	63.7 (10.8)
	G-index	0.7 (0.16)	0.77 (0.17)	0.54 (0.14)

**Table 4:** SVM accuracy, sensitivity, specificity, F1-score, and G-index for high vs. low-medium classification considering different feature sets including full signal (FS), which was the processed signal of IMU (inertial measurement units), 16 features from that signal (FT16), the output of balance board (Wii), and finally HADS (Hospital Anxiety Depression Scale) and TSK (Tampa Scale of Kinesiophobia) questionnaire data (ADT). The reported numbers are in the format of the mean (standard deviation) of the ten runs.

	FS	FT16	Wii	ADT	FS + FT16	FS + Wii	FS + ADT	FT16 + Wii	FT16 + ADT	Wii + ADT	FS + FT16 + Wii	FS + FT16 + ADT	FS + Wii + ADT	FT16 + Wii + ADT	ALL
Accuracy	75.4 (4.2)	66.9 (5.4)	39.5 (8.3)	54.8 (6.8)	63.5 (5.6)	65.2 (6.6)	66.6 (6.3)	58.9 (5.1)	52.7 (7.4)	46.8 (4.8)	64.8 (5.5)	66.8 (6.2)	64.2 (4.9)	54.9 (6.2)	59.3 (5.4)
Sensitivity	72.5 (3.8)	58.3 (5.1)	40.4 (8.8)	55.7 (6.9)	65.8 (5.5)	61.6 (7.9)	67.4 (8.7)	52.1 (5.5)	42.5 (8.6)	48.2 (5.3)	58 (4.9)	68.4 (6.7)	64 (6.1)	44.7 (7.7)	61.5 (4.8)
Specificity	78.2 (5.3)	75.7 (6.5)	38.6 (9.8)	53.9 (7.3)	61.2 (7.3)	68.7 (5.5)	65.7 (7.7)	63.6 (8.9)	62.8 (8.5)	45.3 (6.9)	71.6 (6.4)	65.1 (7.4)	64.4 (5.5)	65.1 (7.6)	65.4 (6.7)
F1-score	74.6 (14.4)	63.9 (13.1)	40.0 (11.5)	55.2 (13.5)	64.3 (14.6)	63.9 (14.9)	66.8 (15.9)	55.3 (16.6)	47.3 (17.3)	47.5 (19.6)	62.2 (11.7)	67.3 (14.4)	64.1 (15.4)	49.8 (14.4)	62.7 (12.3)
G-index	0.35 (0.09)	0.48 (0.12)	0.86 (0.19)	0.64 (0.14)	0.52 (0.13)	0.50 (0.13)	0.47 (0.16)	0.60 (0.14)	0.68 (0.17)	0.75 (0.12)	0.51 (0.11)	0.47 (0.14)	0.51 (0.12)	0.65 (0.15)	0.52 (0.12)

**Table 5:** Statistical analysis on demographic data for all combinations of 2-class classification.

	Low vs Medium-High			Medium vs Low-High			High vs Low-Medium		
	L	MH	*p*-Value (t-Test)	M	LH	*p*-Value(t-Test)	H	LM	*p*-Value(*t*-Test)
**Age [year]**	43.7 (8.2)	43.5 (6.3)	0.86	43.4 (5.6)	43.7 (7.7)	0.84	43.6 (7.3)	43.6 (6.8)	0.97
**Height [cm]**	172.4 (8.3)	172.8 (6.8)	0.75	173.7 (6.7)	171.9 (7.6)	0.24	171.5 (6.9)	173.1 (7.4)	0.34
**Weight** **[** **kg** **]**	80.3 (12.5)	79.2 (12.6)	0.71	80.5 (12)	79.2 (13)	0.56	77.5 (13.3)	80.5 (12.3)	0.31
**BMI [** **kg/m^2^]**	26.9 (3)	26.5 (3.6)	0.56	26.7 (3.8)	26.6 (3.2)	0.91	26.2 (3.4)	26.8 (3.5)	0.48
